# Unveiling Disparities in Beer Consumer Behavior and Key Drivers Across Regions in China

**DOI:** 10.3390/foods14213799

**Published:** 2025-11-06

**Authors:** Jiang Xie, Yiyuan Chen, Ruiyang Yin, Xin Yuan, Liyun Guo, Dongrui Zhao, Jinyuan Sun, Jinchen Li, Mengyao Liu, Baoguo Sun

**Affiliations:** 1 China Food Flavor and Nutrition Health Innovation Center, Beijing Technology and Business University, Beijing 100048, China; 2 Technology Center of Beijing Yanjing Beer Co., Ltd., Beijing 101300, China; 3 Beijing Laboratory of Food Quality and Safety, Beijing Technology and Business University, Beijing 100048, China; 4 Key Laboratory of Brewing Molecular Engineering of China Light Industry, Beijing Technology and Business University, Beijing 100048, China; 5 School of Food and Health, Beijing Technology and Business University, Beijing 100048, China

**Keywords:** beer, consumers, sensory preference, consumer behavior

## Abstract

Beer consumption behaviors within China exhibited significant regional heterogeneity. To elucidate the specific differences in beer consumer behaviors across different regions and their influencing factors, this study systematically analyzed the sensory preference characteristics of consumers in the Chinese beer market based on machine learning methods, and further revealed the core driving mechanisms influencing their consumption behaviors. By integrating consumer data from different regions, a comprehensive dataset was constructed encompassing sensory attribute evaluations (bitterness, malt flavor, hop aroma, smoothness of mouthfeel, foam characteristics, etc.) and other dimensional consumption behavior variables (brand, beer packaging, etc.). Utilizing an ensemble learning framework (LightGBM), Support Vector Machine (SVM), and decision tree models for feature mining, the study identified important factors influencing the consumption behaviors of Chinese beer consumers. Specifically, consumers in mature and upgrading markets placed greater emphasis on the overall drinking experience and drinkability when purchasing beer, whereas consumers in scale-dominant and mainstream competitive markets considered foam persistence, fineness, and light brown color as core quality indicators. Conversely, consumers in potential growth and emerging cultivation markets demonstrated strong brand orientation. This indicated that the factors influencing beer consumption behaviors varied significantly across regions. Through a data-driven paradigm, this study revealed the underlying regional mechanisms behind consumption decisions in different regional beer markets in China, providing a theoretical foundation and empirical support for cross-regional product customization, precision marketing, and resource optimization.

## 1. Introduction

Beer has a long history dating back 13,000 years [1], and is an iconic beverage primarily brewed from malt, hops, and other raw materials through processes including boiling, saccharification, cooling, and fermentation [2]. By now, beer had become a widely favored alcoholic drink among consumers worldwide [3,4]. In 2024, global beer production reached 187.5 billion liters, with the market valued at US$671.54 billion [5,6]. China, as the largest producer, yielded 34 billion liters of beer, accounting for over 15% of the global total production [6]. Driven by the continuing diversification of beer market demand—a trend propelled by both corporate product innovation strategies and consumers’ growing pursuit of flavor variety—the beer market is undergoing a notable structural transformation, as exemplified by the substantial expansion in the availability of craft beer varieties in recent years (a phenomenon described as “hyper-differentiation”), along with a rising consumer expectation that products closely align with their individualized preferences and needs [7,8,9]. This trend was manifested through the growing popularity of craft beers, fruit-flavored beers, beer distillates, and the emergence of new products such as non-alcoholic beers [10,11,12,13], all of which contributed to major market shifts [14]. Thus, understanding beer consumers’ sensory preferences became essential in this process. Furthermore, significant differences were observed in beer consumption markets across various regions in China [15,16,17]. However, insufficient research on cross-regional consumer behavior substantially hindered the expansion of breweries and industry progress. Consequently, there was an urgent need to investigate beer consumption behavior patterns across different Chinese consumption regions, in order to assist breweries in developing region-specific products and formulating targeted business strategies.

Among the numerous factors influencing beer purchasing behavior, sensory preferences (including taste, olfaction, and visual appeal) played a pivotal role, as consumers placed a high priority on consistent quality and specific sensory attributes in their chosen products [18]. Consequently, an increasing number of studies concentrated on analyzing beer consumers’ sensory preferences, establishing relatively mature frameworks. For instance, Natalja Ivanova et al. [19] presented 100 UK beer consumers with different beer samples (*n* = 18) for evaluation. Using the “Rate-All-That-Apply (RATA)” technique, they assessed consumers’ overall liking, perceived mouthfeel, and intensity of consumer-derived attributes. The study found that alcohol content, bitterness, and aroma significantly impacted overall preference. Perla A. Ramos-Parra et al. [20] invited 50 beer consumers for sensory evaluations, including hedonic assessments. Their research emphasized the critical role of beer bitterness in understanding consumer acceptance. Michiel Schreurs et al. [21] analyzed over 180,000 consumer reviews to accurately predict complex food attributes and consumer appreciation. However, these studies predominantly focused on specific regions and examined consumer behavior solely within localized contexts. Substantial evidence demonstrated that sensory perception often varied across geographical and cultural backgrounds [22,23]. As consumer markets grew increasingly complex, research confined to specific regional consumers proved inadequate, with its limitations becoming progressively evident. Consequently, there was an urgent need to investigate beer sensory preferences across different regions through a comparative analysis of nationwide beer consumption markets in China. Such research would facilitate the identification of key sensory factors influencing consumer behavior across various regions and provide actionable insights for breweries to develop strategies aligned with market demands in China.

Previous research on alcohol consumption behavior primarily focused on the intake volume of alcoholic beverages (e.g., beer). However, growing evidence suggested that assessing drinking consumption behavior should be based on consumption patterns rather than simply measuring average intake [24], which required a multidimensional evaluation incorporating factors such as consumption contexts and channels [25]. In the contemporary beer market, consumer behavior has undergone a significant evolution, marked by a shift in purchasing decisions from being primarily driven by price to increasingly emphasizing quality attributes. This transition has been further shaped by a combination of factors such as sensory preferences, packaging appeal, and brand perception [26,27,28]. However, due to the inherent complexity of these factors, traditional methods could not accurately analyze their impact, resulting in unclear understanding of their relative influence magnitude and specific mechanisms. Compared with conventional statistical approaches, machine learning as a computational algorithm possessed feature extraction, data mining, and modeling capabilities with multiple advantages: greater proficiency in handling complex nonlinear relationships, higher efficiency in processing large-scale datasets, more comprehensive evaluation metrics, and enhanced flexibility and precision during model optimization [29]. This study leverages machine learning to investigate beer consumer behavior patterns. This data-driven approach allows for the precise identification of key factors driving consumption, enabling enterprises to implement targeted product enhancements.

**Figure 1:**
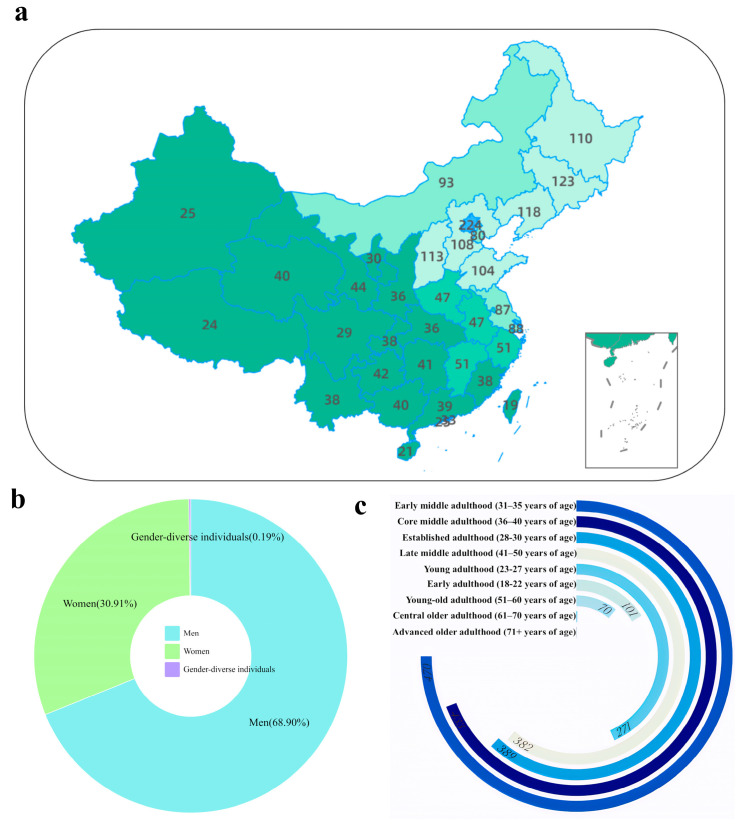
Source and basic information of the questionnaire data. (**a**) Collection sites of the questionnaire data in China. (**b**) Gender distribution of the respondents. (**c**) Age composition of the respondents.

In this study, data were collected from beer consumers across all regions of China using questionnaire surveys (as shown in Figure 1), aiming to achieve the following research objectives: (1) employing quantitative methods to collect data on consumers’ sensory preferences regarding beer color, aroma, and mouthfeel (a complex sensory characteristic resulting from the interaction of haptic, tactile, trigeminal sensations, and temperature-induced stimuli in the oral cavity, associated with the physical and chemical properties of the stimulus) across different regions, while also encompassing other factors influencing consumption behavior, such as brand and packaging; (2) identifying distinct consumption behavior characteristics among demographic groups across regions; (3) utilizing machine learning to determine key factors driving consumption patterns; and (4) constructing a comprehensive descriptive model of beer consumption by integrating behavioral determinants and characteristic patterns.

## 2. Materials and Methods

### 2.1. Questionnaire Design

The online study comprised four modules: (1) beer consumer profiling, (2) beer consumption patterns, (3) sensory dimension evaluations and preference ratings, and (4) questionnaires on factors influencing consumption behavior. Supplementary Materials detailed the sections, domains, and items of the questionnaires. Part 1 assessed consumer demographics through key questions including: “Your place of residence (specifying provincial administrative region)”, “Your occupation and professional status”, “Your daily dietary taste preferences”, and “Your educational attainment”. Additionally, consumer profiles were stratified not only by region but also by age, income, and occupation, enabling separate statistical analyses across demographic segments. Part 2 surveyed consumption frequency of alcoholic beverages (including beer), with detailed quantification of expenditure amounts. To eliminate potential bias in the research results caused by regional income disparities, this study employed a standardized approach by using the ratio of monthly beer expenditure to total monthly income as a metric to classify beer consumption levels into standardized categories. Part 3 evaluated sensory preferences encompassing appearance, packaging, and color characteristics. Following Laureati et al.’s methodology [30], consumer reluctance toward beer off-flavors was assessed using a shortened version of the Italian Food Neophobia (FN) scale, comprising 2 items. Although the original scale has been validated, the modified version was tailored to the specific context of beer off-flavors. To ensure the validity and reliability of the abbreviated scale, a pilot test was conducted with a subset of participants, and internal consistency was evaluated. Part 4 employed an adapted version of the Food Choice Questionnaire (FCQ), originally developed by Steptoe et al. and later modified by Rivaroli et al. for beer products [9], to identify key selection drivers and their correlations with beer attitudes. The FCQ in this study covered four domains: (a) health (2 items), (b) sensory appeal (6 items), (c) packaging (3 items), and (d) brand (2 items). Certain factors from the original adapted FCQ—such as weight control and online convenience—were excluded to enhance focus on attributes more relevant to the current research objectives [31]. The modification process aimed to maintain content validity, and the revised instrument was pretested to ensure its appropriateness for the target population. A unified scoring system was applied to all rated questions: a score of 90–100 was assigned to the most preferred option, 60–70 to the second most preferred, 30–40 to the third, and 0–10 to the fourth.

### 2.2. Sample Information

Data collection was conducted from 31 October 2024, to 1 April 2025, through an online questionnaire administered via the Questionnaire Star platform (https://www.wjx.cn/), targeting respondents aged eighteen years and above. The required sample size was determined using the Raosoft online calculator (http://www.raosoft.com/samplesize.html), with a confidence level of 95%, a margin of error of 5%, and a response distribution set at 50% based on preliminary survey results. The calculation yielded a minimum required sample size of 385. Routine quality control measures were implemented to ensure data integrity and representativeness. To encourage conscientious participation, an incentive measure was introduced whereby several lucky winners were randomly selected from Chinese respondents who completed the questionnaire and awarded beer tasting sessions and a brewery tour experience provided by the National Academy of Liquor Research at Beijing Technology and Business University. The sample covered all regions of China (as shown in Figure 1). Data were subsequently screened by comparing them with results from a second study conducted in these regions. The screening process adhered to two exclusion criteria: removal of individuals who reported “never drinking beer” to ensure relevance of tasting experience, and elimination of records that failed embedded attention or logic checks. This process ensured that the dataset met quantitative and structural requirements for subsequent empirical modeling and established a robust foundation for in-depth analysis. A total of 2119 questionnaires were collected through online channels, among which 2082 were valid responses. Prior to conducting further statistical analyses, we employed multiple established methods to evaluate the reliability and validity of the sensory rating scale [32,33].

### 2.3. Distribution Characteristics of Consumption Influencing Factors Across Different Population Groups

To characterize the distribution patterns of influencing factors, this study primarily employed descriptive statistical methods. First, demographic variables (such as gender, age, and education level) were described using frequencies and percentages to illustrate the basic composition of the sample, and the population sample was categorized accordingly based on these labels. Second, continuous or ordinal-scale influencing factors (such as sensory preference scores and drinking frequency) were summarized using measures including mean and standard deviation to analyze their central tendency and dispersion. All analyses were performed using SPSS 25.0, and the results were presented in the form of tables and charts to comprehensively and clearly display the distribution characteristics of various influencing factors.

### 2.4. Construction of a Consumer Behavior Model and Factors Influencing Consumption

Utilizing collected sensory preference and consumer behavior data, we employed eleven machine learning methods to develop predictive models. Prior to modeling, a comprehensive data cleaning pipeline was implemented to ensure data quality. Invalid responses—including those with uniform answers, implausible response times, or more than 10% missing values—were removed, accounting for approximately 5.2% of the original dataset. Missing values for continuous variables were imputed using k-nearest neighbors (k = 5), while categorical variables were filled with the mode of corresponding features. Outliers were detected using the Interquartile Range (IQR) method applied to sensor ratings and behavioral continuous variables; values falling below Q1 − 1.5 × IQR or above Q3 + 1.5 × IQR were replaced with the variable-specific Winsorized limits, affecting roughly 3.8% of the remaining samples.

To ensure methodological rigor and prevent data leakage, all preprocessing steps—including cleaning, feature engineering, and scaling—were strictly confined to the training set. Feature selection was first performed using high-dimensional LASSO (Hi-LASSO) [34] exclusively on the training data. Subsequently, StandardScaler was fitted on the training set and then applied to transform both the training and test sets. The dataset was partitioned using a stratified sampling strategy (stratify = labels), allocating 30% of the data to the test set to maintain consistent class distribution [35]. To address class imbalance in beer sensory preference data, the synthetic minority over-sampling technique (SMOTE) was applied during the training phase—only to the training set—to generate synthetic minority samples and balance the class distribution. Furthermore, nested cross-validation was implemented for robust model selection and hyperparameter tuning, ensuring unbiased performance estimation and enhancing the generalizability of our results.

Subsequently, eleven representative machine learning classification algorithms—including Random Forest, Naive Bayes, LightGBM, XGBoost, CatBoost, K-Nearest Neighbors (KNN), Gradient Boosting Decision Tree (GBDT), AdaBoost, Support Vector Machine (SVM), Decision Tree, and Extra Trees—were systematically utilized for model training and comparative screening to identify the most suitable model for beer sensory preference prediction. A diverse set of machine learning algorithms with distinct functionalities was selected in order to capture potential patterns in the data from multiple perspectives, thereby enhancing the robustness and generalizability of the results. Furthermore, to ensure a comprehensive and robust evaluation of model performance in this specific classification task, multiple evaluation metrics were deliberately selected based on their complementary strengths: the Area Under the ROC Curve (AUC) was used to assess the overall ranking performance and discriminative ability across different probability thresholds; Accuracy provided an intuitive measure of the correctness of predictions; Precision quantified the reliability of positive predictions; Recall (Sensitivity) evaluated the model’s capability to identify all relevant positive instances; and the F1-score was employed to balance the trade-off between Precision and Recall, which is particularly important in scenarios with class imbalance. This multi-faceted set of metrics was adopted to avoid over-reliance on any single performance indicator and to facilitate a systematic comparison of the generalizability and practical applicability of different machine learning models [21]. Given that the sample size was significantly smaller than the number of features, the data were standardized and cross-validation was employed to avoid overfitting [36]. To rigorously explore the influence of feature variables on the decision-making process of the beer sensory preference prediction model in accordance with the Explainable AI (XAI) paradigm adopted in this study, we employed the SHAP (SHapley Additive exPlanations) algorithm for global interpretability analysis. Specifically, the SHAP open-source library was used to compute mean absolute Shapley values—calculated exclusively on the held-out test set—for each feature across all eleven machine learning models. This approach objectively quantifies the contribution and direction (positive or negative influence) of each feature toward the model’s predictions, enabling a scientifically grounded ranking and evaluation of feature importance. By deriving explanations from unseen test data, this method enhances the validity and trustworthiness of the interpretation. The analysis not only reveals key predictive factors and their operational mechanisms, thereby improving model transparency, but also provides a reliable foundation for subsequent model refinement and knowledge discovery. The entire machine learning workflow, including data preprocessing, model training, hyperparameter tuning (where applicable), and performance evaluation, was completed in the Jupyter Notebook (Version v7) interactive development environment using Python 3.9, with major reliance on the powerful Scikit-learn library for implementing various classification algorithms; meanwhile, the computation of SHAP values required for model interpretability analysis was efficiently performed using the dedicated SHAP library, ensuring consistency and reproducibility of the methodology [37]. The specific code and partial data can be found in the database: https://github.com/jiangjiang12jiang/Questionnaire, accessed on 3 November 2025.

### 2.5. Statistical Analysis and Statistical Methods

Data analysis was performed using multiple statistical methods selected according to research objectives. Data collection was conducted using Wenjuanxing (Enterprise Standard Edition; Changsha Ranxing Information Technology Co., Ltd., Changsha, Hunan, China). Data cleaning and preprocessing were completed in Python 3.9, including handling missing values (mean imputation for continuous variables; separate category/mode imputation for categorical variables) and outlier management using IQR method with winsorization. Reliability of multi-item scales was assessed using Cronbach’s α, and composite scores were generated for analysis. Group comparisons employed one-way ANOVA with Duncan’s post hoc test in SPSS Statistics (Version 25.0; IBM Corp., Armonk, NY, USA), while Spearman’s correlation was used for non-parametric associations. Additional analyses and visualizations were performed using Origin 2024 and R (v3.4.3). Statistical significance was set at *p* < 0.05. This analytical framework ensured appropriate methodology alignment with research questions.

## 3. Results

### 3.1. Sample Description

#### 3.1.1. Background Information and Sample Structure

The demographic characteristics of the sample exhibited broad coverage and a balanced structure (Figure 1). In terms of gender, male respondents accounted for 68.90%, females for 30.91%, and 0.19% identified as non-binary. Figure 1 provides detailed demographic information of the study participants.

The age range spanned from 18 to over 71 years, demonstrating extensive coverage and inclusivity (Figure 1c). Respondents aged 31 to 40 formed the core group, constituting 42.58% of the total sample. The overall distribution approximated a normal distribution, meeting the ideal sampling requirements for consumer behavior research. Among them, the 31–35 age group accounted for the highest proportion (22.18%), followed by the 36–40 age group (20.40%). These age ranges typically represent peak economic and social activity stages, thus exhibiting higher purchasing power and brand engagement [38].

The educational background of the respondents exhibited a multi-tier distribution pattern. Their educational levels covered a spectrum from primary school to postgraduate education, with 59.47% holding at least a bachelor’s degree (51.49% bachelor’s degree and 7.98% postgraduate or higher), 23.36% possessing associate degrees, and 17.17% having received only secondary education or below.

To address geographic variation in income and consumption levels, the income data collected from the questionnaires in this study were first tested for normality using the Shapiro–Wilk test. The results revealed a significant departure from normality (*p* < 0.001), with the distribution exhibiting pronounced positive skewness. Specifically, a large majority of respondents reported incomes within low- and middle-income ranges, while a considerably smaller proportion belonged to the high-income category. This skewed distribution demonstrated high empirical validity, as it not only reflected the socioeconomic profile of the studied population but also aligned with broadly documented patterns of income distribution in empirical socioeconomic research—consistent with expectations underpinned by Pareto or log-normal distributions. These findings suggest that the sample adequately captured the actual income distribution of the general population without introducing substantial systematic bias. Subsequently, based on the ratio of subjects’ income to their monthly beer expenditure, participants were categorized into high-spending (20%), medium-spending (60%), and low-spending (20%) groups.

#### 3.1.2. Reliability and Validity Test

After preliminary data processing, the final questionnaire comprised 2082 valid responses with 29 items, divided into four sections: (1) beer consumer profiling, (2) beer consumption patterns, (3) sensory dimension evaluations and preference ratings, and (4) factors influencing consumption behavior. Five multi-dimensional sensory rating scales were selected for reliability and validity analysis: Q8 (four sensory dimensions), Q14 (beer foam characteristics), Q19 (beer packaging attributes), Q24 (beer brand perceptions), and Q29 (comprehensive influencing factors). Exploratory factor analysis showed that all items had factor loadings above 0.60 and communalities greater than 0.40. The cumulative variance explained by single-factor solutions exceeded 40% for each scale (Q8 = 56.49%, Q14 = 51.30%, Q19 = 44.79%, Q24 = 44.46%, Q29 = 46.54%). The KMO statistics ranged from 0.765 to 0.947, and Bartlett’s tests of sphericity were significant (*p* < 0.001), confirming the suitability for factor analysis and satisfactory discriminant validity [38]. Internal consistency, assessed using Cronbach’s α, met or exceeded the 0.70 threshold for all scales (Q8 = 0.742; Q14 = 0.810; Q19 = 0.823; Q24 = 0.750; Q29 = 0.895), with three scales surpassing 0.80, indicating good to excellent reliability. Overall, these results demonstrated strong reliability and construct validity, providing a robust measurement foundation for subsequent correlation analyses and model estimation.

#### 3.1.3. Overview of Beer Consumer Behavior

The drinking frequency data further reinforced the moderate consumption pattern observed among the participants. Specifically, 20.91% of respondents reported consuming beer 2–3 times per week, and 15.01% indicated consumption once per month or less. Combined, these two groups—representing low to moderate consumption—accounted for 70.75% of the total sample (Table A1). It is worth noting that 37 individuals were identified as never having consumed alcohol; to maintain analytical relevance and accuracy, these respondents were excluded from subsequent analyses.

During the investigation into beer consumption scenarios, it was observed that participants’ drinking behaviors revealed two primary tendencies. The largest segment (39.55%) was characterized by “regular yet controlled drinking habits,” where consumers maintained a stable drinking frequency while exercising moderation in per-session consumption, reflecting a pattern of rational consumption. Another significant group (38.93%) exhibited a preference for “frequent participation in social drinking gatherings or high standards for beer quality” (Table A2). This demographic placed greater emphasis on the social attributes and sensory experience of beer, often consuming it in group settings or on specific occasions. Together, these two consumption patterns collectively delineated a key landscape in the current beer market centered around regularity, moderation, and social orientation.

To account for regional disparities in income and consumption levels, participants were categorized into high-consumption (top 20%), medium-consumption (middle 60%), and low-consumption (bottom 20%) groups based on the ratio of their income to monthly beer expenditure. In the segmentation analysis of high-frequency beer consumers, the data indicated that the most demographically significant group in terms of purchasing power consisted of males aged 31–35, followed by females aged 35–40, who also exhibited considerable consumption capacity. This cohort (31–40 years old) is typically characterized by both ongoing economic upward mobility and a life stage marked by frequent social activities, which together contribute to their heightened purchase intention and increased consumption frequency of beer. Further analysis revealed that this demographic typically had stable income sources and tended to incorporate beer consumption into daily social, entertainment, and leisure scenarios, making beer their alcoholic beverage of priority choice. This consumption characteristic provided clear target audiences and strategic directions for beer companies’ targeted marketing and product positioning.

### 3.2. Statistical Analysis of Factors Influencing Beer Consumer Behavior

#### 3.2.1. Analysis of Consumer Sensory Preferences

**Figure 2:**
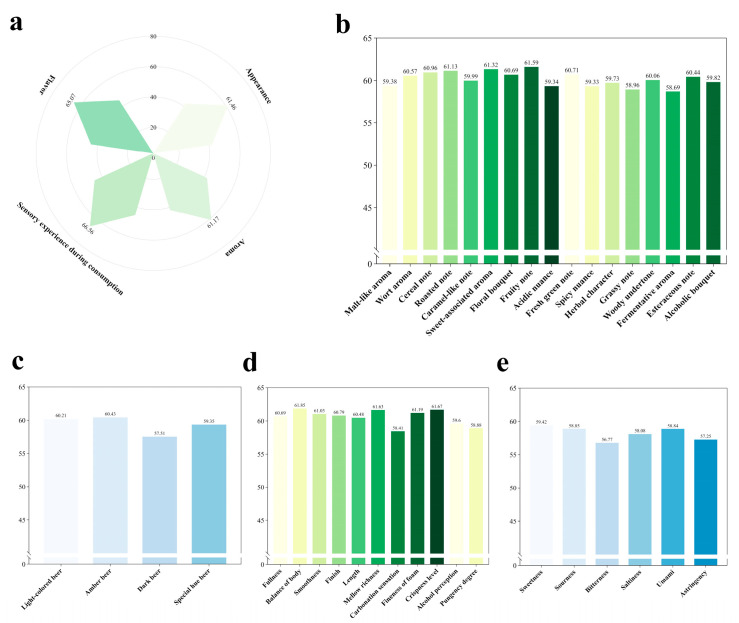
Scores of beer across different dimensions. (**a**) Overall ratings of beer’s “appearance,” “aroma,” “taste,” and “mouthfeel.” (**b**) Specific scores for different “aroma” attributes of beer. (**c**) Specific scores for different “color” attributes of beer. (**d**) Specific scores for beer’s “mouthfeel.” (**e**) Specific scores for different “taste” attributes of beer.

Participants rated their sensory preferences for beer—including color, aroma, taste, and mouthfeel—based on their degree of liking (with the most preferred rated 90–100 points, the second preferred 60–70, the third 30–40, and the fourth 0–10). “Mouthfeel” was the most frequently cited priority in beer purchasing (composite mean score = 66.56), referring to the overall oral sensation during drinking, including smoothness, carbonation (prickling sensation), fullness, and balance. Further analysis revealed that balance (the harmony and integration of various flavor elements) was the most valued aspect of mouthfeel among consumers. Additionally, foam creaminess (the texture of foam in the mouth) was also a notable factor in “mouthfeel” (Figure 2).

This study found that beer consumers also highly valued “taste” in their purchase decisions (composite average score = 65.07). Taste encompasses basic flavor experiences such as sweetness, sourness, bitterness, saltiness, and umami. Among these, “sweetness” received the highest average rating (Figure 2e). Furthermore, the results indicated that preference for “umami” increased with decreasing consumer age. This trend may be attributed to the role of umami in enhancing a sense of relaxation and pleasure during consumption [39], a quality particularly valued among younger consumers.

In the analysis of aroma preferences, ratings were collected for malt aroma, cereal notes, sweet aromas, fruitiness, and fermentation-derived scents, among others. Results indicated that among all aroma attributes, fruit aromas (such as berry, melon, tropical fruit, stone fruit, currant, dried fruit, citrus, red berry, sweet fruit, and green fruit notes) were the most valued by beer consumers, with a composite mean score of 61.59.

In the study on beer color, consumer preferences for beer hues—including light-colored, amber, dark, and special hue—were investigated. Among these, amber beer received the highest score, with a mean rating of 60.43. Notably, in Li et al.’s study on sensory evaluations of different beers, color preference demonstrated a relatively high correlation with overall preference rankings [40]. However, in the present study, consumers placed less emphasis on beer color in comparison. A possible explanation for this discrepancy is that the data collection in our study relied on questionnaire responses rather than direct visual assessment of actual beer samples.

In the investigation of drinking experience preferences, among all the attributes studied, “balance of body” was the most valued aspect by consumers, achieving a high mean score of 61.85.

Notably, differences in the most valued sensory attributes were revealed across age groups and regions. For example, as age increased, consumer focus shifted from appearance to taste and mouthfeel. In terms of regional differences, consumers from southern Chinese provinces (such as Guangdong, Shanghai, etc.) demonstrated a stronger preference for sweetness and fruit aromas, those from northern provinces (such as Beijing, etc.) valued malt character and fullness more highly, while consumers from northwestern provinces (such as Shaanxi) placed greater emphasis on cereal notes.

Furthermore, an analysis was conducted to assess consumers’ aversion to common off-flavors in beer, including phenolic, fatty, and metallic notes. Among these, fatty off-flavors—such as those reminiscent of vegetable oil, rancidity, diacetyl, isovaleric acid, and cheesy aromas—were the least tolerated by consumers (composite mean score = 61.81). This heightened sensitivity may be attributed to typical beer consumption contexts, such as accompanying meals, where consumers often use beer to cut through rich or heavy flavors, thereby increasing their awareness of and aversion to fatty off-notes.

Calculated results showed significant correlations between reported beer purchasing priorities and “color” (*p* < 0.001), “taste” (*p* < 0.001), “mouthfeel” (*p* < 0.001), and “aroma” (*p* < 0.001). Additionally, for taste-driven respondents, aside from the above factors, “the amount of beer foam” was also an important factor influencing purchase behavior. Most respondents preferred fine and creamy foam (described as smooth, creamy, silky, melting softly in the mouth without large bubbles, and contributing to a pleasant drinking experience), a preference particularly pronounced among female participants.

#### 3.2.2. Assessment of Other Factors Shaping Consumer Behavior

**Figure 3:**
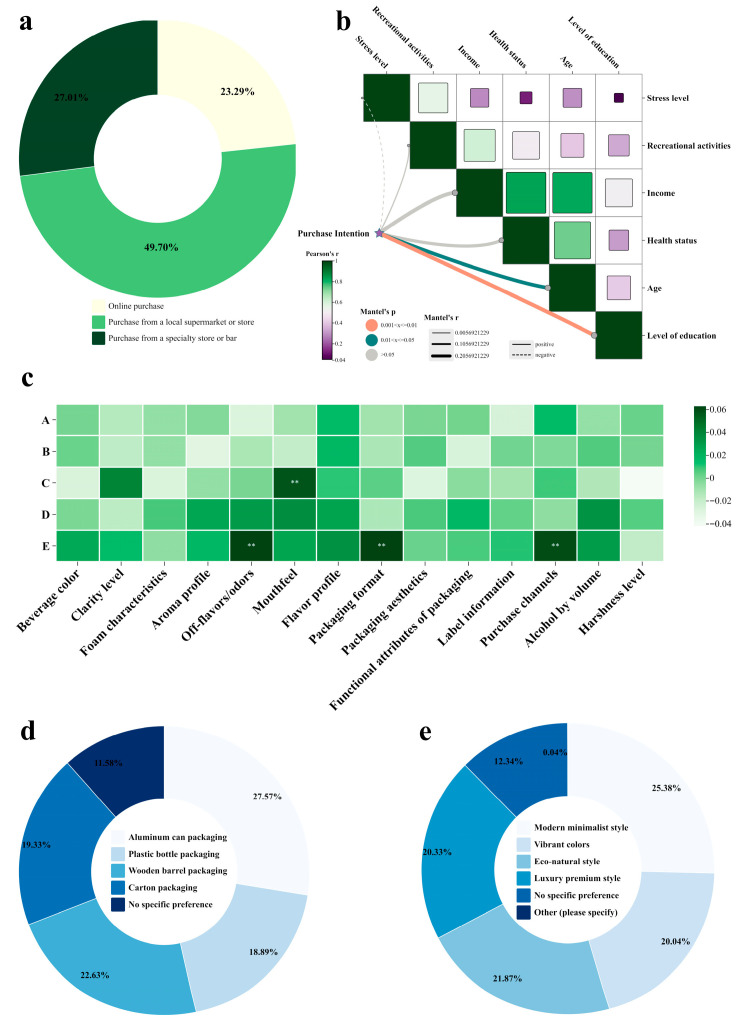
Correlation analysis of factors influencing consumer behavior. (**a**) Proportion of beer purchasing methods. (**b**) Mantel test analysis of the correlation between beer purchase intention and subjects’ own characteristics. The size of the cube is proportional to the magnitude of the Pearson correlation coefficient. (**c**) Heatmap of the correlation between beer brand type ratings and factors affecting consumer preferences. The color gradient represents the strength and direction of the Pearson correlation coefficients, with warmer colors (dark green) indicating positive correlations and cooler colors (light cyan) indicating negative correlations. A represents preference for brands with traditional cultural backgrounds, B represents preference for innovative brands, C represents preference for environmentally friendly or sustainable brands, D represents preference for high-end or luxury brand images, and E represents preference for youthful and dynamic brand images. ** indicate statistical significance at *p* < 0.01. (**d**) Proportion of respondents’ preferred beer packaging. (**e**) Proportion of respondents’ preferred beer packaging styles.

In addition to the aforementioned sensory preferences influencing consumers’ beer consumption behavior, other factors were also found to impact their purchasing decisions. It is worth noting that the study on beer brands did not focus on any specific brand but rather provided a broad characterization—examining what traits of brands appealed to consumers while disregarding the brand identities themselves. From a consumer demographic perspective, age and education level exhibited significant correlations with the factors prioritized when purchasing beer (Figure 3b), which aligns with previous research [41]. Data analysis indicated that age exerted a substantial influence on consumers’ decision-making priorities. Specifically, the 20–30 age group placed significantly greater emphasis on “price” compared to other age groups (adjusted standardized residual AR = 3.3, *p* < 0.01). Meanwhile, this demographic demonstrated significantly higher acceptance toward non-alcoholic and low-alcohol beer products (AR = 2.1, *p* < 0.05). In contrast, respondents aged 55–64 tended to prioritize “location convenience” as a key factor (AR = 2.2, *p* < 0.05). Additionally, the influence of educational background on consumption behavior was observed as follows: In China, consumers with higher education (bachelor’s degree or above) generally exhibited a preference for internationally recognized brands and niche craft beers. This tendency might stem from their association of beer consumption with lifestyle and social identity. They typically demonstrated strong interest in international brands, craft beers, and distinctive flavors, showing willingness to pay a premium for high-quality products with brand narratives.

Furthermore, the study on beer brands revealed that respondents who exhibited higher brand awareness and stronger brand loyalty showed a significantly higher tendency to prioritize “taste” (AR = −3.0). This interesting phenomenon could be interpreted as the consistent and distinctive flavor profiles of established brands attracting and retaining loyal consumers. Additionally, consumers who expressed a preference for beers from specific countries or regions (e.g., Germany, Japan, Belgium demonstrated stronger emphasis on purchasing channels and exhibited greater sensitivity to beer off-flavors (Figure 3c). This tendency may be attributed to their heightened trust in the traditional brewing techniques and quality standards represented by these origin-specific brands. They tended to purchase through reliable channels to ensure product authenticity, while their sensitivity to off-flavors stemmed from strict expectations regarding traditional flavors; any deviation was perceived as a quality defect.

In the contemporary beer market, online purchasing has gained significant popularity and become a major distribution channel. However, in this study, respondents ranked their beer purchasing channel preferences as follows: first, brands available in local supermarkets or stores; second, those obtainable at specialty stores or bars; and third, brands accessible online (Figure 3a). This pattern may be attributed to the predominant consumption contexts of beer consumers, with beer primarily being consumed in dining settings. Only a small segment of beer enthusiasts reported considering online purchases for their preferred brands. Consequently, consumers predominantly favored brands available in local supermarkets or stores, largely due to considerations of convenience.

In the comprehensive investigation of beer packaging preferences among consumers, canned beer was consistently identified as the most preferred packaging format (Figure 3d). This preference was particularly prominent in specific consumption scenarios, including outdoor activities, instant consumption occasions, and modern retail environments. Concurrently, modern minimalist packaging design emerged as the most favored esthetic style, receiving notable endorsement from 57.57% of surveyed participants. Further analysis revealed that the preference for canned packaging was largely driven by its functional advantages, including superior portability, higher transportation safety, better light barrier properties, and enhanced recycling convenience. The appeal of modern minimalist design was primarily attributed to its visual clarity, brand information prominence, and alignment with contemporary esthetic trends in the beverage industry.

### 3.3. Predictive Analysis of Factors Influencing Beer Consumer Behavior

#### 3.3.1. Development of a Machine Learning-Based Consumer Behavior Model for Beer

In the aforementioned study, we conducted an empirical investigation into the factors influencing beer consumers’ purchasing behavior. However, traditional statistical approaches demonstrated limitations in precisely delineating the individual contributions of each factor to overall consumption patterns. To address this gap, machine learning techniques were employed to identify and analyze the key determinants driving the consumption behavior of beer drinkers across diverse regions. Specifically, eleven classification models were developed to discriminate among distinct consumption behavior patterns. Beer consumers were segmented into three categories—high, moderate, and low consumption—based on the ratio of monthly beer expenditure to monthly income. Additionally, prior to modeling, based on prior research, beer consumers across Chinese provinces were categorized into three distinct sets [42]. This regional categorization was established through a multi-dimensional analytical framework that integrated key indicators such as total beer retail sales and market growth rates. This methodology transcended the limitations of single-metric approaches to accurately reflect fundamental regional disparities in beer consumption and the phased evolution of China’s consumer market. Based on this framework, the beer consumption market was segmented into three categories. Mature and upgrading markets: including Shanghai, Beijing, Tianjin, Zhejiang, Jiangsu, Guangdong, Hong Kong, and Macao. These regions were characterized by high disposable income and strong demand for premium products. Scale-dominated and mainstream competitive markets: encompassing Shandong, Henan, Hebei, Jiangxi, Heilongjiang, Jilin, Hubei, Shaanxi, Anhui, Fujian, Hainan, Sichuan, Taiwan, Chongqing, Liaoning, and Hunan. This segment featured large population bases and represented the most intensely competitive arena for mainstream consumers. Potential-growth and emerging cultivation markets: covering Yunnan, Qinghai, Guizhou, Tibet, Guangxi, Gansu, Ningxia, Shanxi, Inner Mongolia, and Xinjiang. These areas were identified by their rapid growth potential and early-stage market development characteristics.

**Figure 4:**
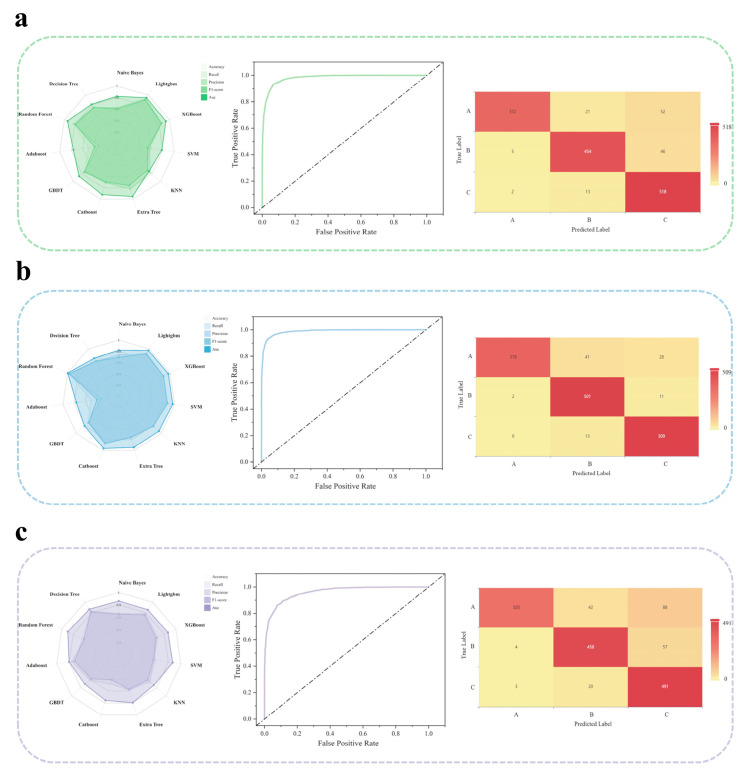
The predictive performance of machine learning models was evaluated using multiple quantitative metrics. For classification models, the metrics are defined as follows: Accuracy reflects the proportion of correct predictions overall; Recall measures the model’s ability to identify positive instances; Precision represents the proportion of true positive cases among those predicted as positive. Higher values for these three metrics indicate better model performance. The F1-score is defined as the harmonic mean of Precision and Recall and is suitable for evaluating imbalanced datasets. The Area Under the Curve (AUC) reflects classification efficacy, with a value closer to 1 indicating superior performance. (**a**) Radar chart of the eleven evaluated models in the mature and upgrading markets, along with the ROC curve and confusion matrix heatmap of the optimal model. (**b**) Radar chart of the eleven evaluated models in the scale-dominated and mainstream competitive markets, along with the ROC curve and confusion matrix heatmap of the optimal model. (**c**) Radar chart of the eleven evaluated models in the potential-growth and emerging cultivation markets, along with the ROC curve and confusion matrix heatmap of the optimal model.

The classification performance was evaluated using AUC, F1-score, accuracy, recall, and precision (Figure 4a–c), with no signs of overfitting observed (Figure 4). Based on these five metrics, LightGBM, Support Vector Machine (SVM), and Decision Tree were selected as the optimal models to construct analytical models for the factors influencing consumer behavior in the mature and upgrading markets, scale-dominated and mainstream competitive markets, and potential-growth and emerging cultivation markets, respectively (Figure 4). Analysis of consumer behavior prediction models across different market segments revealed notable disparities in the predictive efficacy of machine learning algorithms (Figure 4). In the prediction task for consumer behavior in mature and upgrading markets, the LightGBM model achieved the best overall performance (AUC = 0.896, Accuracy = 84.7%), with its AUC value being significantly higher than that of the baseline model (Figure 4a). For modeling consumer behavior in scale-dominated and mainstream competitive markets, the Support Vector Machine (SVM) outperformed other models across five key metrics—accuracy, precision, recall, F1-score, and AUC (Figure 4b)—attaining an AUC of 0.976 and a classification accuracy of 91.9%. In predicting consumer behavior in potential-growth and emerging cultivation markets, the Decision Tree model emerged as the optimal choice (Figure 4c), achieving an AUC of 0.882 and an accuracy of 85.9%. It was noteworthy that in the parallel investigation into the graded models, the average AUC of the eleven machine learning models exceeded 0.6 (Figure 4), further validating the general applicability of the methodology. Synthesizing the results from the above cross-regional studies, it was demonstrated that the machine learning approach exhibited strong robustness (Robustness) in classifying consumer behaviors across different cultural backgrounds, with its superior performance being consistently confirmed across diverse geographic population datasets and varied modeling tasks.

#### 3.3.2. Application of SHAP-Based Feature Analysis to Conduct In-Depth Research on Key Factors Driving Consumer Behavior Using Machine Learning Methods

Based on the accurate analysis of key factors influencing beer consumers’ purchasing behavior, this study not only enhanced the efficiency and scientific rigor of food research but also established a methodological framework for correlating multidimensional influencing factors of beer with consumer behavior. LightGBM, Support Vector Machine (SVM), and Decision Tree were selected as the optimal predictive models for identifying influencing factors of consumer behavior in three distinct beer consumption markets in China, respectively (Figure 4a–c). Specifically, three different analytical approaches were employed to identify key factors affecting consumer behavior. First, feature importance metrics were quantified to evaluate the contribution of each variable within the predictive models. Second, SHapley Additive exPlanations (SHAP) values were applied to interpret model outputs at a local level, elucidating the influence mechanism of individual features on the prediction of specific sample attributes [23]. This approach provided profound insights into the multivariate relationships between beer characteristics and consumer responses, thereby effectively identifying the primary drivers behind the predictions. Ultimately, through the integrated application of these methods, a core set of beer attributes closely associated with consumer behavior was successfully identified.

**Figure 5:**
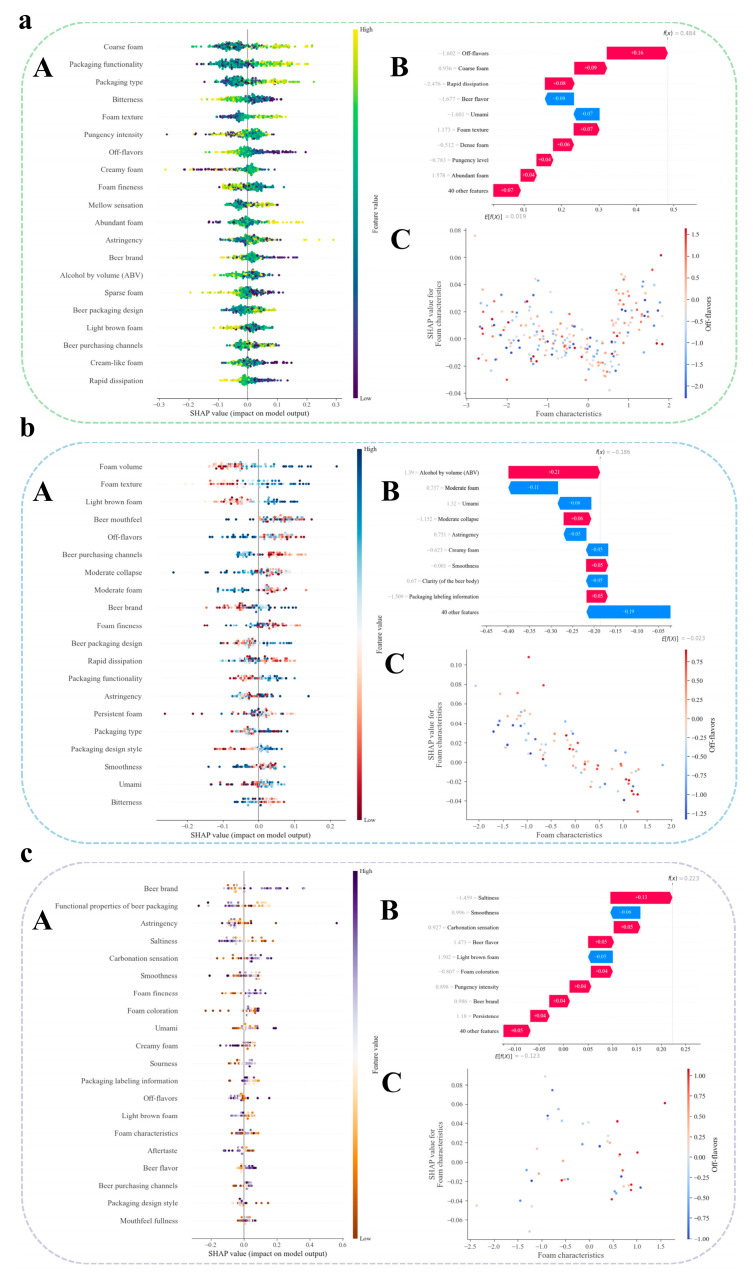
Identification of core influencing factors based on beer consumers’ purchasing behavior. (**a**) SHAP summary plot for consumer samples from mature and upgrading markets regarding purchasing behavior influences. (**b**) SHAP summary plot for consumer samples from scale-dominated and mainstream competitive markets regarding purchasing behavior influences. (**c**) SHAP summary plot for consumer samples from potential-growth and emerging cultivation markets regarding purchasing behavior influences. (**A**) The SHAP summary plot displays the top 16 features influencing purchasing behavior in consumer samples based on the classification model. Each point in the plot represents a sample in the dataset. The color gradient indicates the feature values. The horizontal axis shows the direction and magnitude of each feature’s impact on the model predictions: positive SHAP values indicate an enhancing effect, while negative values indicate a suppressing effect. For example, if high-value data points cluster in the positive x-axis region, it indicates that an increase in the concentration of this feature significantly enhances the model predictions. (**B**) Feature contribution plot for a single prediction instance. Values represent the SHAP contribution of each feature to the model output. Features are ranked by the magnitude of their contribution. Positive values increase the prediction score, while negative values decrease it. (**C**) SHAP Dependence Plot of the Feature. This figure depicts the relationship between the value of the feature (x-axis) and its impact on the model output, represented by the SHAP value (y-axis). Each point in the plot represents an individual instance from the dataset. The trend line illustrates how the contribution of the feature to the model prediction changes as its value varies. A positive SHAP value indicates that the feature increases the prediction, while a negative value signifies a decrease in the prediction.

In the context of the mature and upgrading markets, key influencing factors were identified accordingly. Compared with consumers in other market segments, beer consumers in these markets demonstrated greater emphasis on beer packaging, particularly in terms of both “packaging style” and “packaging functionality.” This trend may have been attributed to the distinctive purchasing habits and underlying socio-psychological factors specific to these markets [43]. For consumers in these markets, relatively low ratings for “bitterness” in beer positively influenced their purchasing behavior (Figure 5a). This finding suggested that while consumers in mature and upgrading markets appreciated moderate bitterness in beer, exceeding a specific threshold inhibited purchasing behavior—a result consistent with previous studies [44]. Additionally, beer foam emerged as a significant factor affecting purchasing decisions in these markets. Whereas consumers in other market segments exhibited a distinct preference for “fine foam,” those in mature and upgrading markets showed a stronger inclination toward “coarse foam.”

Furthermore, regional analysis of the scale-dominated and mainstream competitive consumer markets indicated that consumers in these markets attached significant importance to the quality characteristic of “foam” in their sensory evaluation of beer. This concern extended beyond the mere presence of foam to stringent requirements for its multidimensional physical attributes: consumers particularly valued sufficient and persistent foam volume (head retention), a fine and creamy foam texture, and foam coloration exhibiting a typical light brown hue—a visual characteristic often regarded as an indicator of the authenticity of specific beer varieties and malt properties. Compared to consumers in other markets, those in the scale-dominated and mainstream competitive markets demonstrated notably heightened sensitivity and concern regarding “off-flavors” in beer. These off-flavors referred to negative sensory characteristics that deviated from the expected beer profile, potentially originating from raw material quality issues (e.g., oxidized or light-struck notes), fermentation by-products (such as diacetyl, acetaldehyde, or sulfidic compounds), or deficiencies in process control (e.g., sterilized or metallic flavors).

In the study of beer consumers in potential-growth and emerging cultivation markets, it was found that consumers in these markets placed considerable emphasis on beer brands. This heightened attention to branding was likely shaped by specific market conditions, consumer culture, and the marketing ecosystem. Furthermore, consumers in these markets paid greater attention to the “astringency,” “salinity,” and “umami” of beer. These differences in sensory preferences may have stemmed from dietary habits in the regions comprising these markets, which tended toward high-fat and high-salt foods.

## 4. Discussion

### 4.1. Analysis of Beer Consumer Characteristics Across Different Regions in China

Beer consumption behavior is dynamic and variable, shaped by a wide array of demographic and socioeconomic factors—including age, gender, income, and regional background—exhibiting considerable heterogeneity across different consumer segments. Constructing well-defined consumer profiles is essential for deciphering market structures, facilitating targeted marketing strategies, and informing data-driven product development.

This section provided a comprehensive overview of the demographic characteristics of Chinese beer consumers across different age groups: The younger generation (18–30 years old), as an emerging consumer force. Brand and packaging are critical factors in attracting them. Their consumption scenarios are more diverse, including social gatherings with friends and online interactions.

The middle-aged generation (30–50 years old) constitutes the main consumer base for premium and craft beer. With relatively strong purchasing power, they are willing to pay a premium for quality, brand appeal, and unique drinking experiences. They are both consumers of traditional beer and central figures in exploring new styles, valuing factors such as ingredients and sensory experiences.

The older generation (50 years and above) exhibits relatively traditional and stable consumption habits. While less sensitive to price, they demonstrate high brand loyalty. They typically form a stable consumer group for traditional industrial lager beers, preferring familiar tastes and brands. Their consumption scenarios are mostly centered around family gatherings and business entertainment.

As one of the world’s oldest alcoholic beverages, beer has developed distinct consumer markets across different regions of China. Beer consumption increasingly exhibited characteristics of regional variation, while premiumization and rejuvenation had emerged as cross-regional market drivers. Consumer preferences were gradually transitioning from mass-produced industrial beers toward craft beers, distinctive flavors, and high-quality experiences. This shift not only reflected a broader trend of consumption upgrading but also highlighted the ongoing integration and innovation within Chinese beer culture.

### 4.2. Analysis of Factors Influencing Beer Consumption Behavior Among Different Beer Consumer Markets

#### 4.2.1. Beer Sensory Dimensions

Chinese beer consumption was facing dual challenges of regional cultural specificity and divergent sensory preferences, a phenomenon that exhibited systematic differences across different markets. An in-depth examination of the underlying mechanisms driving these differences was of considerable theoretical and practical value for advancing the understanding of contemporary Chinese beer consumption behavior and informing data-driven product development and marketing strategy optimization.

Consumer preference weighting for beer sensory attributes exhibited notable regional variations. Systematic research in the selected regions revealed that consumers in mature and upgrading markets prioritized mouthfeel as the most valued sensory dimension, far exceeding other attributes. This finding challenged the traditional notion that taste or aroma dominated beer selection [40]. Specifically, consumers in mature and upgrading markets demonstrated a unique sensory preference structure ordered as follows: mouthfeel > appearance > taste > aroma. This experience-driven hierarchy reflected that consumers in emerging mature and upgrading markets placed greater emphasis on the integrated sensory experience of beer, rather than on isolated sensory attributes.

In studies conducted in selected regions, consumers in scale-dominated and mainstream competitive markets demonstrated a more differentiated preference pattern. They displayed strong loyalty to traditional flavor characteristics such as balanced bitterness, floral and fruity aromas, while simultaneously placing greater emphasis on taste purity and exhibiting heightened sensitivity to “off-flavors” in beer. The relatively low tolerance for undesirable flavors among beer consumers in these markets may have originated from the region’s mature tasting culture and the resulting stringent internal quality benchmarks.

Beer consumption preferences in potential-growth and emerging cultivation markets exhibited a markedly distinct pattern compared to other regions. Specifically, consumers in these markets placed greater emphasis on sensory attributes such as astringency, salinity, and umami in beer. This divergence in preferences may have stemmed from multiple factors: on one hand, the unique dietary culture in the regions comprising these markets—particularly the prevalent consumption of high-salt, fermented, and umami-rich foods—may have shaped consumers’ sensitivity and expectations regarding flavor characteristics; on the other hand, the relatively younger age demographic of beer consumers in potential-growth and emerging cultivation markets may have driven a pursuit of more intense, complex, and heterogeneous flavor profiles, which may have reflected a growing inclination among the new generation toward products with distinct personality and sensory layers [45].

Within China, the depth of consumers’ cognitive understanding of beer sensory attributes and their preference structures underwent distinct stages of evolution as drinking frequency increased. For instance, low-frequency consumers primarily relied on overall mouthfeel impressions as their selection basis; medium-frequency consumers developed heightened sensitivity to negative attributes (such as off-flavors or bitterness); while high-frequency consumers ultimately acquired the ability to identify and pursue subtle pleasurable characteristics. This evolutionary trajectory of sensory cognition revealed an experience accumulation curve in the beer domain, which provided a new theoretical perspective for market segmentation.

#### 4.2.2. Other Factors

The present study identified systematic differences among beer consumers in selected Chinese markets, revealing that their distinct consumption behavior patterns essentially resulted from the co-evolution of regional geographical characteristics, intrinsic product attributes, sensory experience standards, and consumers’ psychological determinants.

The study demonstrated that consumers in potential-growth and emerging cultivation markets placed considerable emphasis on beer brands. This tendency not only reflected specific market environments and cultural factors but also highlighted consumers’ psychological reliance on brands as cognitive shortcuts in decision-making processes. When confronted with increasingly abundant product choices and growing market homogenization, consumers in these markets frequently used brands as cognitive anchors to identify product origins, differentiate style categories, and comprehend brewing philosophies. Consequently, brands functioned not only as identifiers but also evolved into psychological symbols conveying trust, quality assurance, and emotional reassurance—thereby reducing consumers’ perceived risk and enhancing their purchase confidence.

Simultaneously, the study found that consumers in mature and upgrading markets attached greater importance to beer packaging, including its style design and information presentation. This trend was closely associated with the local cultural context: in these market regions, beer was often consumed as a gift or in group social settings; therefore, whether the packaging appeared dignified and suitable for gifting or public presentation became a significant factor influencing consumer decision-making. Moreover, younger demographic groups increasingly perceived visually appealing and conversation-stimulating packaging as an expression of personal identity and lifestyle. This psychological driver transformed packaging into a medium for self-expression and social signaling. This dimension of emotional resonance prompted brands to treat packaging not only as a functional carrier, but also as a strategic tool for value communication and emotional connection.

#### 4.2.3. Analysis of Key Beer Consumer Groups Across Regions

Mature and upgrading markets underwent a critical phase of rapid transformation and consumption upgrading, characterized by a notable younger demographic shift in core consumer groups. Predominantly composed of Millennials, this consumer cohort demonstrated distinct preferences for personalization and exhibited heightened discernment regarding quality. During their beer selection process, they prioritized sensory characteristics such as flavor and taste, while simultaneously attaching significant importance to packaging design and overall consumption experience. They demonstrated clear preferences for product packaging, tending to choose beer products featuring modern minimalist styles, eco-friendly materials, and interactive design elements (such as recyclable cans). Meanwhile, in terms of sensory experience, this consumer group placed particular emphasis on the holistic drinking experience, including smoothness of body, flavor complexity, balanced bitterness, and clean finish—reflecting a behavioral shift from “drinking” to “savoring” beer.

The core consumer demographic in potential-growth and emerging cultivation markets remained centered around young and middle-aged adults. This group demonstrated distinct brand awareness in their beer consumption preferences, typically favoring beer brands with high recognition and a strong reputation. Simultaneously, they displayed strong interest and a particular preference for craft beers, emphasizing diversity in flavor profiles, brewing techniques, and regional characteristics. The trend toward rational drinking emerged as a significant driving force in these markets, prompting a shift in consumer preferences toward health consciousness and moderate alcohol consumption. This shift, in turn, substantially stimulated growth in demand for low-alcohol and non-alcoholic beer products. Additionally, convenience became a major factor influencing purchasing decisions, with packaging formats such as canned beer—suited for various ready-to-drink scenarios—gaining increasing market popularity.

The main consumer demographic in scale-dominated and mainstream competitive markets was primarily composed of younger populations and the middle class. Influenced by regional culture, this group demonstrated heightened sensitivity to off-flavors in beer, which were generally perceived as quality issues and significantly diminished their purchase intention. Such consumption behaviors not only reflected the stringent quality requirements among consumers in these markets, but also indicated their acute sensory discrimination capabilities cultivated through cultural accumulation and experiential familiarity.

Beer held far greater significance in human society than merely being a beverage, as it embodied cultural identity and historical memory. This study yielded multiple implications for the beer industry’s practice. In terms of marketing, enterprises were advised to develop differentiated strategies for various market segments, such as highlighting sensory experiences and design esthetics in mature markets, while strengthening brand storytelling and cultural resonance in growth markets. In product design, it was necessary to optimize the product portfolio based on consumer group characteristics, including developing low-alcohol products aligned with health trends, creating craft beer series with innovative sensory experiences, and designing convenient packaging suitable for ready-to-drink scenarios.

The research not only provided practical guidance for the beer industry but also expanded the academic horizons of consumer behavior theory, sensory science, and cross-cultural studies. Future studies were recommended to transcend geographical limitations, enhance longitudinal tracking, and promote deeper integration of neuroscience, genetics, and cultural studies to construct a more systematic theoretical framework for beer sensory preferences. The empirical theoretical system established by this study offered a scientific basis for targeted product development in specific markets, sensory quality optimization, and culturally adaptive marketing strategies, thereby facilitating sustainable development and innovative prosperity for the beer industry in the era of consumer sovereignty.

### 4.3. Limitations and Future Directions of the Research

Although this study yielded valuable insights, several limitations must be acknowledged, particularly concerning the representativeness of the sample and the generalizability of the findings. Additionally, the use of an online convenience sampling method constrained the external validity of the results. The study participants primarily represented digitally active segments of the Chinese population, which may have limited the broader applicability of the findings to the general public. Furthermore, the geographical restriction of the sample to selected regions of China reduced the cultural and socioeconomic diversity of the respondents. Consequently, populations with limited internet access—such as some elderly, rural, and low-income groups—were underrepresented, further compromising the study’s external validity. Although the research design intentionally incorporated participants from diverse age groups and consumption backgrounds, the sampling strategy may still not have fully captured the characteristics of the broader Chinese population. Additionally, in terms of the questionnaire design, it failed to include specific questions directly assessing participants’ perception of alcohol content, although alcohol content represents an important determinant of the perceived characteristics of alcoholic beverages, including beer.

To enhance the generalizability of future research, it is recommended to expand the sample size, employ probability sampling methods, and incorporate participants from more diverse geographical and socio-demographic backgrounds. The questionnaire could also be refined by including items related to cultural background, which would enable a more systematic examination of cultural influences on consumer behavior. Cross-regional or cross-cultural comparative designs, as well as mixed-mode surveys that combine online recruitment with offline methods (such as street-intercept or door-to-door surveys), could further improve the representativeness and robustness of future samples.

## 5. Conclusions

This study employed machine learning methods to analyze rating scales of factors influencing Chinese beer consumers’ purchasing behavior, incorporating Spearman correlation analysis to achieve effective integration of subjective preferences and objective data. Through a systematic analysis of consumer profiles and their influencing factors across different regions, the study revealed the complexity and diversity of beer consumption behaviors among different demographic groups and geographic areas. The main findings were as follows:

First, beer consumption behaviors demonstrate significant demographic and regional characteristics. In terms of age distribution, younger consumers (18–30 years old) act as the primary drivers of flavor innovation and low-alcohol beverage trends; middle-aged consumers (30–50 years old) form the core consumer base for premium and craft beers; while older consumers (above 50 years old) demonstrate the strongest brand loyalty and tend to maintain traditional product preferences. Geographically, mature and upgrading markets placed emphasis on drinking experience and packaging design, while scale-dominated and mainstream competitive markets attached greater importance to traditional flavors and purity quality. Potential-growth and emerging cultivation markets exhibited a dual structure characterized by the coexistence of craft innovation and classic tastes.

Secondly, key factors influencing beer consumption behaviors encompass multiple dimensions such as sensory attributes, brand, and packaging. The study revealed that sensory preferences exhibited distinct regional variations and evolutionary trajectories: among the surveyed consumers, those in mature and upgrading markets placed greater emphasis on the overall drinking experience in their sensory evaluations, whereas consumers in scale-dominant and mainstream competitive markets demonstrated particular sensitivity to off-flavors. Brand and packaging functioned as significant market differentiators. Consumers in potential growth and emerging markets attached considerable importance to beer brands, while those in mature and upgrading markets focused more prominently on packaging design.

This study provided significant actionable guidance for the beer industry. Brewing enterprises were advised to implement targeted strategies across different geographic segments and consumer demographics: focusing on packaging design and taste refinement in mature and upgrading markets, maintaining quality standards and traditional brewing techniques in scale-dominant and mainstream competitive markets, and prioritizing flavor innovation and brand storytelling development in potential growth and emerging cultivation markets. Simultaneously, the industry was recommended to continuously monitor consumption upgrading trends and proactively capitalize on emerging opportunities such as low-alcohol beverages, premium product offerings, and flavor diversification.

## Data Availability

The original contributions presented in the study are included in the article/Supplementary Material, further inquiries can be directed to the corresponding author.

## Supplementary Materials

The following supporting information can be downloaded at: https://www.mdpi.com/article/10.3390/foods14213799/s1, Table S1: Drinking frequency of subjects; Table S2: Drinking context selection.

## Abbreviations

The following abbreviations are used in this manuscript:

NN	neural network
SVM	support vector machines
RF	random forests
DT	decision tree
AUC	area under the ROC curve
MSE	mean squared error
RMSE	root mean squared error
MAE	mean absolute error
MAPE	mean absolute percentage error

## Appendix A

**Table A1:** Drinking frequency of subjects.

Options	Number of People	Proportion
Rarely drink alcohol (only on special occasions per year)	106	5.09%
Occasionally drink alcohol (once a month or less)	318	15.27%
Infrequent drinking (a few times per month)	407	19.55%
Moderate frequency (once per week)	331	15.90%
Regular drinking (2–3 times per week)	443	21.28%
Frequent drinking (almost daily)	189	9.08%
High-frequency drinking (at least once daily)	184	8.84%
Very high-frequency drinking (multiple times daily)	60	2.88%
Constant drinking (almost continuously throughout the day)	44	2.11%

**Table A2:** Drinking  Context  Selection.

Options	Number of People	Proportion
Only on special occasions or occasionally	523	24.68%
Regular but moderate drinking habits with controlled consumption	838	39.55%
Higher drinking frequency, often including social drinking outside	739	34.87%
Frequent participation in gatherings and social drinking, or high standards for alcohol quality	825	38.93%
Purchasing premium alcohol or frequent outings for drinking	376	17.74%
Consumption at upscale bars/clubs or collecting fine alcohols	224	10.57%

Note: This is a multiple-answer question; each respondent may select more than one option.
