# Supplementary material for: Unveiling Disparities in Beer Consumer Behavior and Key Drivers Across Regions in China
Source: Foods. 2025 Nov 6;14(21):3799. doi: 10.3390/foods14213799 (PMC12609601; doi:10.3390/foods14213799)
Supplement: Supplementary file 1 [file foods-14-03799-s001.zip › Supplementary Table captions.pdf]

Unveiling disparities in beer consumer behavior and key driv-ers across regions in China

Supplementary table captions:

Table S1. Drinking frequency of subjects

Table S2. Drinking context selection
